# Multi-Centered Pre-Treatment CT-Based Radiomics Features to Predict Locoregional Recurrence of Locally Advanced Esophageal Cancer After Definitive Chemoradiotherapy

**DOI:** 10.3390/cancers17010126

**Published:** 2025-01-03

**Authors:** Nuo Yu, Xiaolin Ge, Lijing Zuo, Ying Cao, Peipei Wang, Wenyang Liu, Lei Deng, Tao Zhang, Wenqing Wang, Jianyang Wang, Jima Lv, Zefen Xiao, Qinfu Feng, Zongmei Zhou, Nan Bi, Wencheng Zhang, Xin Wang

**Affiliations:** 1 Department of Radiation Oncology, National Cancer Center/National Clinical Research Center for Cancer/Cancer Hospital, Chinese Academy of Medical Sciences and Peking Union Medical College, Beijing 100021, China; yunuohh@163.com (N.Y.);; 2 Department of Radiation Oncology, Jiangsu Province Hospital/The First Affiliated Hospital with Nanjing Medical University, Nanjing 210029, China; 3 Department of Radiation Oncology, Tianjin Medical University Cancer Institution & Hospital, Tianjin 300060, China

**Keywords:** radiomics, esophageal cancer, prediction, recurrence, radiotherapy

## Abstract

**Purpose**: We constructed a prediction model to predict a 2-year locoregional recurrence based on the clinical features and radiomic features extracted from the machine learning method using computed tomography (CT) before definite chemoradiotherapy (dCRT) in locally advanced esophageal cancer. **Patients and methods**: A total of 264 patients (156 in Beijing, 87 in Tianjin, and 21 in Jiangsu) were included in this study. All those locally advanced esophageal cancer patients received definite radiotherapy and were randomly divided into five subgroups with a similar number and divided into training groups and validation groups by five cross-validations. The esophageal tumor and extratumoral esophagus were segmented to extract radiomic features from the gross tumor volume (GTV) drawn by radiation therapists before radiotherapy, and six clinical features associated with prognosis were added. T stage, N stage, M stage, total TNM stage, GTV, and GTVnd volume were included to construct a prediction model to predict the 2-year locoregional recurrence of patients after definitive radiotherapy. **Results**: A total of 264 patients were enrolled from August 2012 to April 2018, with a median age of 62 years and 81% were males. The 2-year locoregional recurrence rate was 52.6%, and the 2-year overall survival rate was 45.6%. About 66% of patients received concurrent chemotherapy. In total, we extracted 786 radiomic features from CT images and the Principal Component Analysis (PCA) method was used to screen out the maximum 30 features. Finally, the Support Vector Machine (SVM) method was used to construct the integrated prediction model combining radiomics and clinical features. In the five training groups for predicting locoregional recurrence, the mean value of C-index was 0.9841 (95%CI, 0.9809–0.9873), and in the five validation groups, the mean value was 0.744 (95%CI, 0.7437–0.7443). **Conclusions**: The integrated radiomics model could predict the 2-year locoregional recurrence after dCRT. The model showed promising results and could help guide treatment decisions by identifying high-risk patients and enabling strategies to prevent early recurrence.

## Simple Summary

This study developed a prediction model to forecast a 2-year locoregional recurrence in patients with locally advanced esophageal cancer who underwent definitive chemoradiotherapy (dCRT). The model combined clinical and radiomics features extracted from pre-treatment computed tomography (CT) images. A total of 264 patients from three centers were included in the study, with clinical features like tumor stage and tumor volume and radiomic features used to construct the model. The Support Vector Machine (SVM) method integrated these features to predict recurrence. In the training group, the model showed excellent performance (C-index: 0.9841), while in the validation group, it demonstrated moderate performance (C-index: 0.744). The prediction model can help in personalizing treatment strategies for esophageal cancer patients, guiding therapy decisions to potentially improve outcomes.

## 1. Introduction

Esophageal cancer is the ninth most common malignancy globally and is the sixth leading cause of cancer mortality [1]. In Asian countries, most cases of esophageal cancer are histopathologically classified as esophageal squamous cell carcinoma (ESCC). With advancements in technology and increased awareness of physical examinations, the prognosis of esophageal cancer has shown improvement, with 5-year overall survival (OS) rates increasing from 16–39% to 33–60% [2,3,4,5]. The 2-year locoregional recurrence rate for definitive chemoradiation is around 26–56% [6,7,8]. Advanced diagnostic technologies have significantly improved the effectiveness of early detection for esophageal cancer; however, further progress is needed to enhance prognostic outcomes in treatment [9,10]. Therefore, individualized stratified therapy is an important means to further improve the prognosis of esophageal cancer. As a result, increasing studies are focusing on prognosis prediction models, including clinical features, radiomics, and gene-based models [11,12,13].

Accurate prognostic evaluation is crucial for tailoring individualized treatment plans and improving patient outcomes of ESCC. Traditional prognostic methods, such as TNM staging and histopathological assessment, often fall short of capturing tumor heterogeneity and predicting patient-specific outcomes. Radiomics, an emerging field that extracts high-dimensional quantitative features from medical imaging, provides a novel solution to these challenges [14,15]. By leveraging routine imaging modalities like computed tomography (CT) and integrating advanced computational techniques, radiomics enables the identification of subtle imaging biomarkers that reflect tumor phenotype, microenvironment, and genetic characteristics.

First proposed by Philippe Lambin in 2012, radiomics is a newly developed technique that utilizes the acquired images from CT, Magnetic Resonance Imaging (MRI), or positron emission tomography (PET) using the advanced computational or deep learning methods to aid in diagnostic staging, response evaluation, and prognostic analysis [16]. Using a radiomics-based prediction model has facilitated the identification of high-risk and low-risk patients, as well as the prediction of early recurrence, distant metastasis, disease-free survival (DFS), and OS in many other tumors like lung cancer or rectal cancer, etc. [17,18,19,20]. In esophageal cancer, studies have also applied radiomics using PET, CT, and other imaging techniques to predict survival [21,22]. Incorporating radiomics has been shown to improve predictive performance, with the AUC value reaching from 0.71 to 0.83 [22].

CT, as the most widely used imaging modality for the diagnosis and treatment evaluation of esophageal cancer, has proven particularly suitable for radiomics analysis. Tang et al. found that the integrated model of CT radiomics and clinical features could predict a one-year recurrence with the area under the curve (AUC) of 0.809, better than that of radiomics or clinical features alone [11]. However, many existing CT radiomics models for predicting recurrence in esophageal cancer lack multicenter validation, limiting their generalizability and accuracy. These limitations underscore the need for further research to enhance the reliability of radiomics-based approaches and integrate them effectively into clinical practice.

Building on these findings, our study aims to leverage multicenter data to develop a predictive model that integrates CT radiomics and clinical features to predict a two-year locoregional recurrence in patients with locally advanced ESCC treated with definitive chemoradiotherapy (dCRT). Furthermore, we seek to evaluate the accuracy and clinical utility of this model, addressing the current limitations of single-center studies and advancing the application of radiomics in this challenging patient population.

## 2. Materials and Methods

### 2.1. Patients

A total of 555 patients with locally advanced ESCC from August 2012 to April 2018 were collected from three institutions in China (212 in Beijing, 257 in Tianjin, and 86 in Jiangsu). Finally, 264 patients (156 in Beijing, 87 in Tianjin and 21 in Jiangsu) who met the eligibility criteria were enrolled: (1) Age ≥ 18 years old; (2) Clinical stages T1-4, N0-1, M0-1B (American Joint Committee on Cancer version 6, M1b was limited to lymph node metastasis in the supraclavicular or celiac trunk area); (3) Unresectable or inoperable due to complications, or refusal of surgery by the patient; (4) Radiation dose EQD2 (Equivalent Dose in 2 Gy/f) ≥ 60 Gy; (5) Minimum follow-up time was 2 years for surviving patients; (6) Treatment with definitive radiotherapy with or without concurrent chemotherapy as the primary treatment; (7) Availability of complete clinical data.

All patients underwent the contrast-enhanced CT (CECT) before treatment to assess the disease and extract features. The locoregional lymph node was considered metastasis when its short axis was greater than 10 mm on CECT according to the 8th AJCC TNM staging system. Gastric endoscopy was also applied to evaluate the location and length of the lesion. Endoscopic ultrasound (EUS) was used to determine the T stage and assess suspicious peritumoral lymph nodes. After a thorough examination, patients received definite chemoradiotherapy. At the end of radiotherapy, the CECT was given again to evaluate the treatment effect and acted as the baseline for evaluating locoregional recurrence later. The clinical data such as sex, age, T, N, M, and TNM stage were also recorded.

### 2.2. Data Preparation

**Figure 1:**
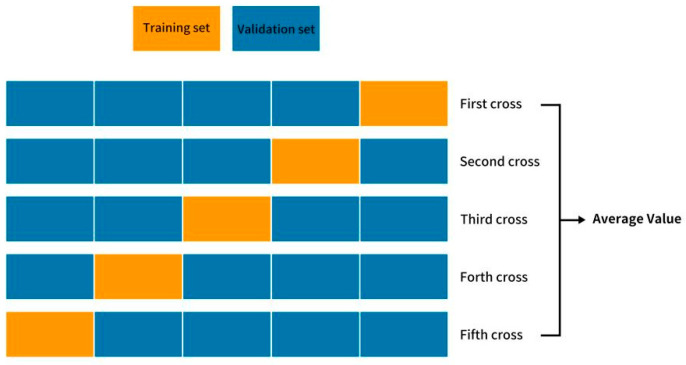
The workflow of a five times repeated random hold-out experiment to divide the training and validation groups.

Considering the unbalanced distribution of censored data, a five times repeated random hold-out experiment was employed [23]. All patients were randomly divided into five subgroups with similar numbers of patients, among which four subgroups were used as the training group and the remaining one was used as the validation group. One of the five subgroups was selected as the validation group each time, thus forming five different validation groups (validation groups 1, 2, 3, 4, 5) and corresponding training groups (training groups 1, 2, 3, 4, 5). The average values were calculated to evaluate the predictive performance of the two groups. Figure 1 illustrates the workflow of this study.

### 2.3. Follow-Up

Follow-up visits were made every 3 months for the first 2 years and every 6 months thereafter. The locoregional recurrence was recorded as the endpoint of the study, and it was defined as a relapse in the esophagus or regional lymph nodes, which were evaluated using cervical, thoracic, and abdominal CT, lymph node ultrasound, or gastric endoscopy, and reconfirmed by biopsy or PET-CT scan. The OS was also recorded, which was defined as the interval between the start date of radiotherapy and the last follow-up or death.

### 2.4. CT Image

Before receiving definite radiotherapy, all patients underwent CECT (Philips Brilliance CT Big Bore, Amsterdam, Netherlands or Siemens SOMATOM Definition AS 40, Erlangen, Germany). The CT scan parameters were as follows: 120 kV; 180 effective mAs; beam collimation of 16 × 1.5 mm; a matrix of 512 × 512; a pitch of 0.813; and a gantry rotation time of 0.75 s. After non-enhanced CT scanning, a dynamic contrast-enhanced CT scan was performed following intravenous administration of 2.0–2.5 mL/s nonionic contrast material (Ioversol Injection, 100 mL, 320 mg/mL, Hengrui, Lianyungang, China) using power injection at a rate of 3 mL/s followed by saline flush (20 mL). Arterial-phase images were obtained 43 s after contrast injection. The thickness of reconstructed slices was 5.0 mm. Arterial-phase CT images were retrieved for image feature extraction.

### 2.5. Volume of Interest (VOI) Segmentation and Feature Extraction

After patients underwent CT simulation scanning, the images were uploaded to the Pinnacle system. CT intensity was normalized to reduce variations caused by different scanning machines and pixel spacing. Radiologists delineated the gross tumor volume (GTV), representing the primary tumor region, and gross tumor volume of metastatic lymph nodes (GTVnd), representing potentially metastatic lymph nodes. Tumor areas were distinguished from normal tissues such as the lung, heart, and spinal cord based on CT images. Radiomic features were extracted from the GTV, and the volumes of GTV and GTVnd were calculated using the Pinnacle system.

The Marching Cubes (MC) algorithm was adopted for 3D data reconstruction. This algorithm efficiently reconstructed high-quality images that accurately reflected the tumor’s shape, which is crucial for developing predictive models for locoregional recurrence in locally advanced esophageal cancer [24].

In this study, we extracted four types of radiomic features from the 3D VOI: (1) Texture features describe surface properties and are resistant to noise, making them robust for matching patterns despite local deviations. (2) Statistical features are derived from histograms based on the probability distribution of pixel intensities. (3) Wavelet feature was used to extract spatial information of 3D images with different frequency components, mainly extracting high-frequency and low-frequency information of 3D images. (4) HOG features focus on edge and shape information by analyzing gradient direction distributions, which reflect the local and spatial gradient characteristics of the 3D image.

### 2.6. Feature Selection

In this study, Principal Component Analysis (PCA) was used to screen out the features [25]. PCA is one of the most widely used data dimension reduction algorithms. The main idea of PCA was to map the n-dimensional features to the k-dimension, which is a new orthogonal feature, also known as the principal component and was a reconstructed k-dimensional feature based on the original n-dimensional features. Compared to methods like LASSO or Recursive Feature Elimination (RFE), PCA was selected for its computational efficiency and its ability to retain the maximum variance in the data, ensuring that key patterns are preserved while reducing redundancy. This made PCA particularly suitable for high-dimensional radiomics data.

PCA algorithm flow: (1) Subtract the mean value of each one-dimensional feature. (2) Calculate the covariance matrix. (3) Calculate the eigenvalues and eigenvectors of the covariance matrix using Singular Value Decomposition (SVD). (4) Select the first 30 principal components by sorting the eigenvalues in descending order to retain the most informative features while excluding redundant or noise-prone dimensions, as they account for the majority of the variance in the dataset. Then, the corresponding 30 feature vectors were used as column vectors, respectively, to form the feature vector matrix. (5) Transform the data into a new space constructed by 30 feature vectors. (6) The first 30 features, namely the principal components after dimensionality reduction, were selected as effective features for the model.

### 2.7. Construction of the Radiomics Model

SVM (Support Vector Machine), XGBoost (eXtreme Gradient Boosting), Logistic Regression, neural networks, and decision tree classification models were constructed for the above-filtered features [26,27,28]. Firstly, the image data were divided into a training set and a validation set, and the training set was used to establish a classifier describing the pre-defined data class. By learning a mapping or function through the training set, the corresponding classification model was established and applied to classify the new data.

By verifying and comparing the accuracy, robustness, and stability of models constructed by different methods, the final modeling method was determined as SVM. SVM was chosen due to its robustness in handling high-dimensional data, ability to model complex non-linear relationships, and demonstrated stability across multiple validation sets. These characteristics make SVM particularly suitable for radiomics applications, where data dimensionality and complexity are high. SVM is a commonly used classification method in machine learning, often applied to pattern recognition, classification, and regression analysis. The classification model of SVM was mainly used for binary classification in this study.

The remaining 30 radiomics features after screening were integrated with prognostic-related clinical features, including T stage, N stage, M stage, total TNM stage, GTV, and GTVnd volume. The SVM method was used to construct the individual radiomics prediction models.

SVM algorithm process:

1. Use training set to build feature X-result Y model:

*D*_1_ = {(*x*^(1)^,*y*^(1)^),(*x*^(2)^,*y*^(2)^),…,(*x*^(*N*)^,*y*^(*N*)^)}

Suppose there were N data in the training set *D*_1_, and each data x (vector) had N features. Y was the category of x and assume there were k categories.

2. Find a hyperplane to divide the dataset into two classes, ensuring that the nearest points to the hyperplane (support vectors) are as far from it as possible.

### 2.8. Prognostic Performance Evaluation

The model evaluation included three aspects: differentiation degree, calibration degree, and clinical practical value. The differentiation was evaluated by the AUC of the ROC (Receiver Operating Characteristic) curve. The calibration degree was evaluated through the calibration curve. The greater the agreement between predicted and observed OS states, the better the calibration of the model. A decision curve, which quantified net benefits across thresholds from 0 to 1 in the validation cohort, was plotted to determine the clinical usefulness of the prognostic model. Higher clinical value was indicated when the decision curve deviated more from the two extremes (treat-all and treat-none).

### 2.9. Statistical Analysis

The extraction of radiomics features and construction of models was accomplished with AIMED (Artificial Intelligence In Medicine, Beijing, China. URL: https://www.blothealth.com/, 2022) and PyCharm (A tool providing intelligent coding assistance, Prague, Munich, and Amsterdam, Europe. URL: https://www.jetbrains.com/pycharm/. Statistical analysis was carried out with the R software version 3.6 (R Core Team. R: A language and environment for statistical computing. R Foundation for Statistical Computing, Vienna, Austria. URL: http://www.R-project.org, 2016) and SPSS version 26.0 (IBM Corp., Armonk, NY, USA). Survival rates were estimated using the Kaplan–Meier method and compared with the log-rank test. Statistical significance was defined as *p* < 0.05.

## 3. Results

### 3.1. Patients’ Clinical Characteristics

The clinical characteristics of all patients are summarized in Table 1. A total of 264 locally advanced ESCC patients were included. The median age of the patients was 62 years (range: 34–84), and 81% were male. Approximately 66% of patients received concurrent chemotherapy, of whom 111 (63.4%) were treated with paclitaxel- and platinum-based regimens. The median follow-up time was 60.0 months, and the median survival time was 18.8 months. The 2-year OS rate and locoregional recurrence rate were 45.6% and 52.6%, respectively. A total of 127 patients developed locoregional recurrence.

### 3.2. Radiomics Features Extraction and Selection

In this study, four types of radiomic features were extracted from 3D VOI. A total of 786 features were extracted from contrast-enhanced CT images, including 540 HOG features, 42 texture features, 48 wavelet features, and 156 statistical features.

Among the 786 radiomics features extracted, 47 were invalid, including 4 infinite values, 15 null values, and 28 values with a variance of 0. The above invalid features were eliminated. From the remaining 739 features, PCA was used to screen out the 30 highest radiomic features related to recurrence status. In this study, five-fold cross-validation was adopted, 30 radiomics features were screened out for each fold cross-validation, and 150 radiomics features were screened out for a total of a five-fold cross.

The distribution of the selected radiomics feature types was as follows: 77 HOG features, 49 statistical features, 16 wavelet features, and 8 texture features, and the detailed descriptions are reported in Supplementary Table S1.

### 3.3. Construction and Evaluation of the Radiomics Model

**Figure 2:**
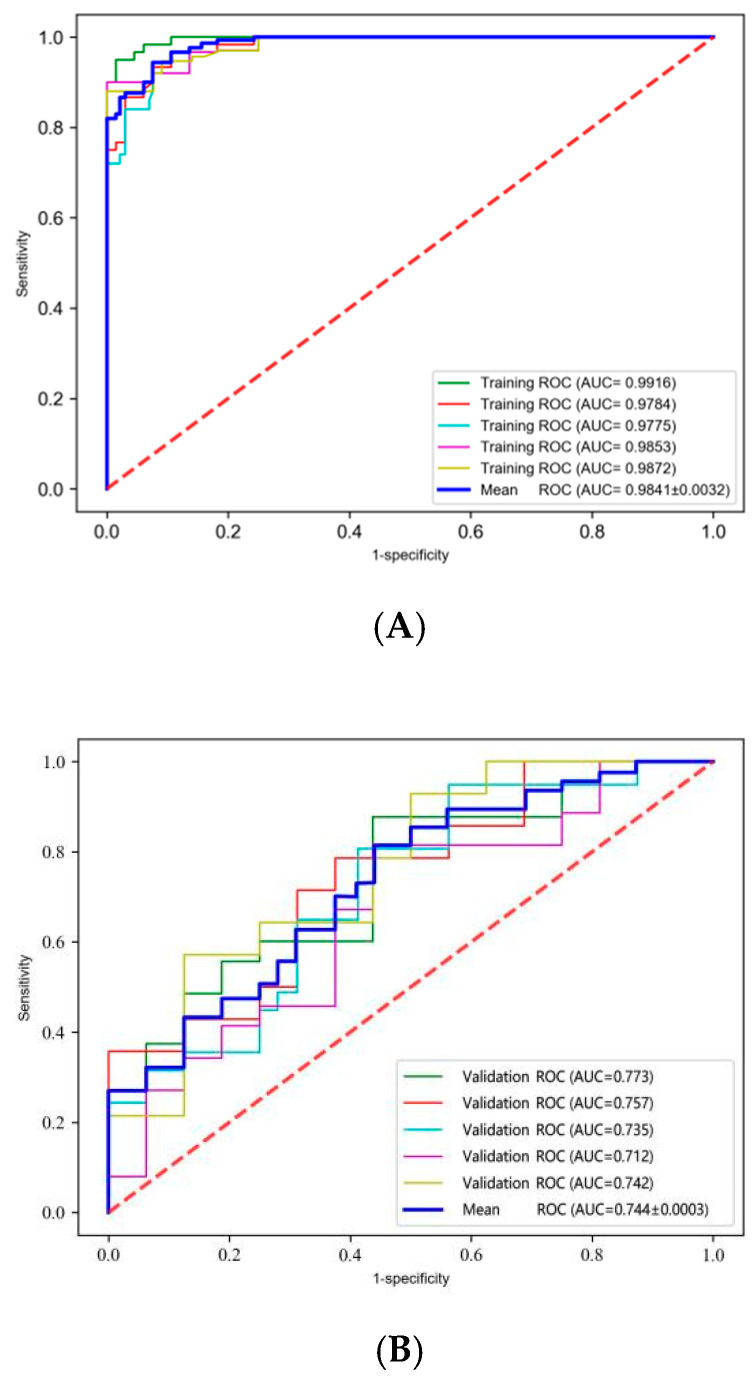
The ROC curve of training groups and validation groups. (**A**) the AUC curves of five training groups, respectively, and the average curve. (**B**) the AUC curves of five validation groups, respectively, and the average curve.

**Figure 3:**
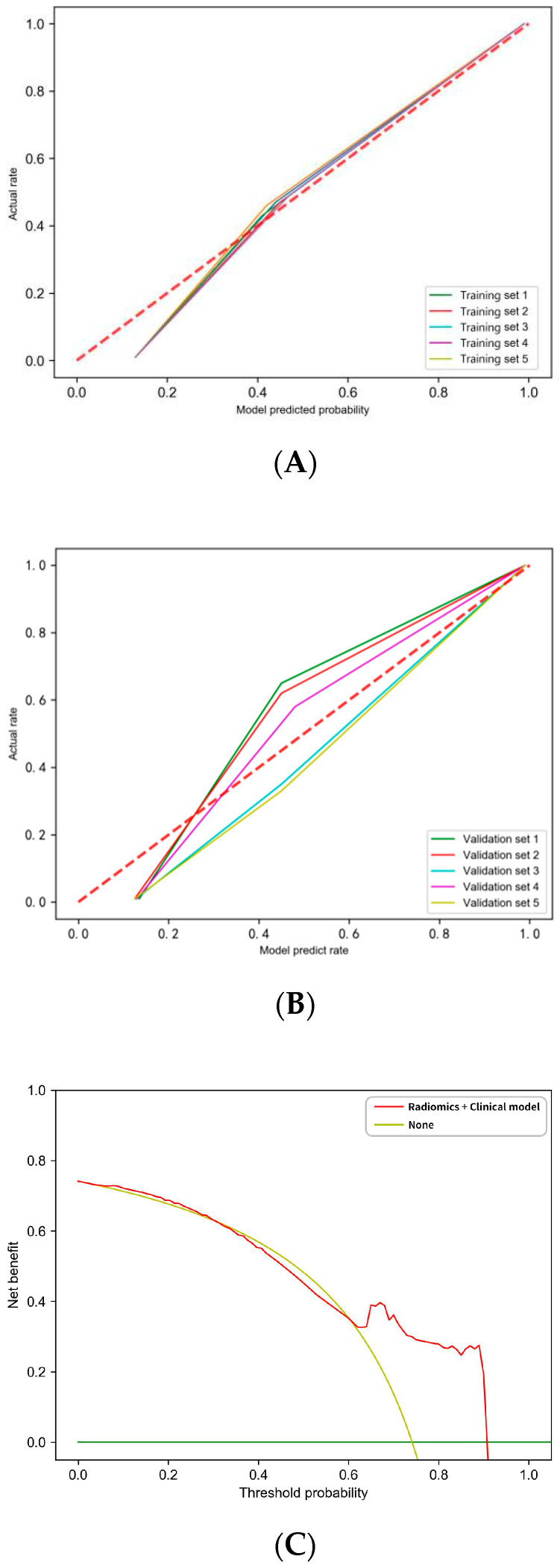
The calibration curves and decision curves. (**A**) the calibration curves of five training groups. (**B**) the calibration curves of five validation groups. (**C**) The decision curve of the radiomics and clinical model. The *x*-axis represents the threshold probability and the *y*-axis represents the net benefit.

Through the SVM method, we constructed the prediction model in every hold-out experiment using 30 radiomics features and six clinical features including T, N, M, and TNM stage, as well as GTV and GTVnd volume. To account for censoring data, five repeated random hold-out experiments were performed to complete the internal validation. For the prediction of the 2-year locoregional recurrence, the detailed AUC values of five cohorts were presented in Table 2. The average AUC value of the model in the training set was 0.9841 ± 0.0032, indicating excellent predictive performance and high discriminative power. The ROC curves for the training and validation sets, including individual folds and their average performance, are shown in Figure 2. The ROC curves of the training set were steep and close to the top-left corner, showing consistent results across the five cross-validation folds. The validation set had an average AUC value of 0.744 ± 0.0003, reflecting a lower predictive capability compared to the training set. The ROC curves of the validation set were less steep and further from the top-left corner, with greater variability between the folds. The average accuracy, sensitivity, and specificity of the two groups are shown in Table 3. The model demonstrated strong performance, with higher accuracy, sensitivity, and specificity in the training set compared to the validation set, reflecting consistency in predictive capability across both datasets. Figure 3 illustrates key aspects of model performance and decision-making utility. In Figure 3A,B, the calibration curves for the five training and validation groups show the agreement between predicted and actual outcomes, indicating the models’ reliability. In Figure 3C, the decision curves show that when the threshold probability exceeds 0.6, patients experience greater net benefit using radiomics and clinical models.

## 4. Discussion

We established a radiomics model to predict the 2-year locoregional recurrence of locally advanced esophageal cancer based on the pre-treatment CT images. Results showed that the training sets had a high predictive performance. All the training groups obtained a high AUC value of 0.97. However, the validation groups did not attain similar prediction efficiency and the average AUC value of all validation groups attained 0.74. The calibration curves showed that our training model fitted the reference line, which indicated the high consistency of the prediction and the real results. The decision curve demonstrated that when the recurrence rate is more than 60%, the model would receive more net benefit in clinical practice.

Based on the prediction model, we have approximately 70% accuracy in predicting whether a patient will experience early recurrence. In other words, when clinicians analyze pre-treatment CT images, there is substantial confidence in predicting local recurrence after chemoradiotherapy, enabling the formulation of appropriate treatment strategies. If patients have a high probability of relapse, more proactive treatments, such as adjuvant therapy or a more intensive follow-up, should be implemented to prevent or detect early recurrence. Strategies for designing the volume of interest in radiotherapy could also be adjusted, such as appropriately expanding the target volume to include more prophylactic irradiation of lymph node drainage areas or utilizing the simultaneous integrated boost method to enhance local control rates for high-risk patients. Additionally, higher dose prescriptions may be more suitable for patients prone to relapse. Many malignant tumors have progressed to the era of precision therapy, and esophageal cancer is moving in the same direction. Radiomics has seen significant advancements in recent years, aiming to stratify patients by risk and predict prognosis before treatment, representing a major step toward personalized therapy. It is evident that, in clinical practice, evaluating the risks of relapse or distant metastasis solely using the AJCC staging system is insufficient. Radiomics, a non-invasive, quick, and simple tool, has demonstrated its ability to effectively distinguish between patients. Moreover, radiomics reuses existing imaging data, conserving significant resources and reducing costs for both clinicians and patients.

In this study, all these results indicated that our radiomics model demonstrated relatively accurate predictive performance compared to other studies [11]. In a similar study reported by Qiu et al., they extracted eight clinical features and seven radiomic features to build an integrated radiomics model to predict a 1-year and 2-year regional recurrence [29]. The C-index of the training group was 0.746 (95%CI, 0.680–0.812) and the test group was 0.724 (95%CI, 0.696–0.752). They achieved a lower AUC value in the training set but obtained similar results in the validation set compared to ours. Currently, the exploration of patient imaging data has expanded beyond radiomics, with deep learning emerging as a significant approach [30,31]. Recent studies have highlighted the value of deep learning-based radiomics combined with other clinical features in constructing predictive models for esophageal cancer prognosis. Notably, these studies reported improvements in predictive performance, with enhanced AUC values compared to traditional radiomics models. In comparison to our study, their AUC values were slightly higher (0.657–0.805 vs. 0.744) in the validation group, with models incorporating deep learning achieving superior predictive performance. Deep learning’s ability to automatically extract complex and high-dimensional features from imaging data makes it a powerful complement to conventional methods. Looking ahead, the integration of deep learning with radiomics, multi-omics data, genomics, and clinical variables is expected to become a key research direction [32]. This approach holds the promise of enhancing the accuracy and robustness of predictive models, paving the way for more precise and personalized treatment strategies.

Compared with other studies, the disparity between the training group and the validation group in our research is notable. The validation set’s average AUC value of 0.744 ± 0.0003 reflects moderate predictive performance, indicating that the model has potential clinical utility in stratifying patients based on risk. However, the lower accuracy in the validation set compared to the training set suggests limitations in the model’s generalizability. The primary reasons for this are as follows: high AUC values can sometimes indicate overfitting. Certain prediction models may perform exceptionally well in the training set but struggle to generalize to validation sets, rendering them ineffective when applied to external data. In our study, we collected imaging data from three centers and included a substantial number of patients to minimize overfitting and enhance the model’s generalizability. However, despite these efforts, the noticeable AUC gap between the training and validation groups suggests that the model still exhibits some degree of overfitting. This may be attributed to the model’s complexity, which could lead to the inclusion of noise and fitting of sampling errors during training. To address this issue, CT data were standardized during image processing, with pixel values normalized by their mean and variance to improve the model’s adaptability across datasets.

Moving forward, simplifying models or using models with appropriate complexity will be essential in mitigating overfitting. Second, the relatively limited number of imaging datasets likely contributes to these challenges. Recruiting more patients, collecting additional CT images for analysis, and including more centers for external validation, could help address this issue. Another potential solution is model regularization, which adds constraints to the objective function to enhance generalizability and optimize performance. We used PCA for feature reduction, but methods like LASSO and RFE could provide more compact and interpretable models. These approaches will be considered in future work to improve model performance and clinical applicability. Patient demographics and inter-center variability could limit the model’s generalizability. Differences in factors like age, sex, and comorbidities may affect outcomes, while variations in clinical practices and imaging protocols across centers could impact data consistency. Future studies should include larger, more diverse cohorts and standardized protocols to improve model generalization. Additionally, emerging techniques such as artificial intelligence and deep learning provide promising strategies to mitigate overfitting [33]. Recent studies on esophageal cancer prognosis prediction have demonstrated that integrating radiomic features with deep learning can significantly improve model performance, resulting in higher AUC values. Future research should explore the application of deep learning to further enhance model robustness and predictive accuracy. Another key limitation of the SVM model used in this study is its lack of interpretability, which restricts insight into the contribution of individual features to predictions. Future studies will explore more interpretable models or integrate techniques like SHapley Additive exPlanations (SHAP) or Local Interpretable Model-Agnostic Explanations (LIME) to enhance transparency and clinical applicability. In addition, in the diagnosis, treatment, and prognosis prediction of esophageal cancer, besides single CT-based radiomics, PET/CT and MRI-based radiomics also play a crucial role. Moreover, the integration of multi-omics data, such as genomics and proteomics, could significantly enhance model construction, providing better tools for prognosis prediction and personalized treatment. Therefore, future efforts should focus on the combined application of multi-omics to address the challenges of modern medicine.

## 5. Conclusions

We developed and validated a multi-center radiomics model to predict the 2-year locoregional recurrence in patients with locally advanced esophageal cancer, incorporating both CT imaging features and clinical data after definitive chemoradiotherapy. Our integrated model shows promising results in predicting locoregional recurrence. In the future, we anticipate that this radiomics-based prognostic model will play a significant role in treatment decision-making by identifying patients at varying risk levels, thereby enabling strategies to prevent early recurrence.

## Figures and Tables

**Table 1:** Baseline characteristics of the 264 patients with esophageal cancer.

Characteristics	Number of Patients (n = 264) (%)
**Gender**	
Male	215 (81.4)
Female	49 (18.6)
**Age (years)**	
Mean	61.61
Range	34–84
**T stage**	
T1	5 (1.9)
T2	29 (11)
T3	91 (34.5)
T4	139 (52.7)
**N stage**	
N0	23 (8.7)
N1	241 (91.3)
**M stage**	
M0	192 (72.7)
M1a	22 (8.3)
M1b	50 (18.9)
**cTNM stage**	
IIA	7 (2.7)
IIB	22 (8.3)
III	163 (61.7)
IVA	22 (8.3)
IVB	50 (18.9)
**GTV**	
Mean	44.69
Range	6.6–172.7
**GTVnd volume**	
Mean	16.4543
Range	0–297.37
**Concurrent chemotherapy**	
Yes	175 (66.3)
No	89 (33.7)
**Local recurrence**	
Yes	127
No	137

**Table 2:** The AUC value of the five hold-out experiment groups, respectively, in the training and validation group. * AUC, Area Under Curve.

Prediction Model	AUC *					
	Group 1	Group 2	Group 3	Group 4	Group 5	Average
Training set	0.9916	0.9784	0.9775	0.9853	0.9872	0.9841 ± 0.0032
Validation set	0.773	0.757	0.735	0.712	0.742	0.744 ± 0.0003

**Table 3:** The average Accuracy, Sensitivity, and Specificity value of the five hold-out experiment groups in the training and validation group.

Prediction Model	Accuracy	Sensitivity	Specificity
Training set	0.946 ± 0.031	0.960 ± 0.029	0.917 ± 0.032
Validation set	0.858 ± 0.018	0.870 ± 0.022	0.833 ± 0.026

## Data Availability

Research data are stored in an institutional repository and will be shared upon request to the corresponding authors.

## Supplementary Materials

The following supporting information can be downloaded at: www.mdpi.com/article/10.3390/cancers16230000/s1, Table S1: Distribution of seleted radiomics features.

## Abbreviations

ESCC	esophageal squamous cell carcinoma
CT	Computer Tomography
MRI	Magnetic Resonance Imaging
PET	positron emission tomography
DFS	disease-free survival
OS	overall survival
AUC	area under the curve
CECT	contrast-enhanced CT
VOI	Volume of interest
GTV	gloss tumor volume
GTVnd	gross tumor volume of metastatic lymph nodes
MC	Marching Cubes
HOG	Histogram of oriented gradient
PCA	Principal Component Analysis
SVD	Singular Value Decomposition
SVM	Support Vector Machine
XGBoost	eXtreme Gradient Boosting
ROC	Receiver Operating Characteristic

## References

[1] Bray F., Ferlay J., Soerjomataram I., Siegel R.L., Torre L.A., Jemal A. (2018). Global cancer statistics 2018: GLOBOCAN estimates of incidence and mortality worldwide for 36 cancers in 185 countries. CA Cancer J. Clin..

[2] Tepper J., Krasna M.J., Niedzwiecki D., Hollis D., Reed C.E., Goldberg R., Kiel K., Willett C., Sugarbaker D., Mayer R. (2008). Phase III trial of trimodality therapy with cisplatin, fluorouracil, radiotherapy, and surgery compared with surgery alone for esophageal cancer: CALGB 9781. J. Clin. Oncol..

[3] Allum W.H., Stenning S.P., Bancewicz J., Clark P.I., Langley R.E. (2009). Long-term results of a randomized trial of surgery with or without preoperative chemotherapy in esophageal cancer. J. Clin. Oncol..

[4] Shapiro J., van Lanschot J., Hulshof M., van Hagen P., van Berge Henegouwen M.I., Wijnhoven B., van Laarhoven H., Nieuwenhuijzen G., Hospers G., Bonenkamp J.J. (2015). Neoadjuvant chemoradiotherapy plus surgery versus surgery alone for oesophageal or junctional cancer (CROSS): Long-term results of a randomised controlled trial. Lancet Oncol..

[5] Yang H., Liu H., Chen Y., Zhu C., Fang W., Yu Z., Mao W., Xiang J., Han Y., Chen Z. (2021). Long-term Efficacy of Neoadjuvant Chemoradiotherapy Plus Surgery for the Treatment of Locally Advanced Esophageal Squamous Cell Carcinoma: The NEOCRTEC5010 Randomized Clinical Trial. JAMA Surg..

[6] Wang S.X., Marshall M.B. (2021). Chemoradiation Therapy as Definitive Treatment of Esophageal Cancer. Surg. Clin. N. Am..

[7] Versteijne E., van Laarhoven H.W., van Hooft J.E., van Os R.M., Geijsen E.D., van Berge Henegouwen M.I., Hulshof M.C. (2015). Definitive chemoradiation for patients with inoperable and/or unresectable esophageal cancer: Locoregional recurrence pattern. Dis. Esophagus..

[8] Bedenne L., Michel P., Bouché O., Milan C., Mariette C., Conroy T., Pezet D., Roullet B., Seitz J.F., Herr J.P. (2007). Chemoradiation followed by surgery compared with chemoradiation alone in squamous cancer of the esophagus: FFCD 9102. J. Clin. Oncol..

[9] Chou C.K., Karmakar R., Tsao Y.M., Jie L.W., Mukundan A., Huang C.W., Chen T.H., Ko C.Y., Wang H.C. (2024). Evaluation of Spectrum-Aided Visual Enhancer (SAVE) in Esophageal Cancer Detection Using YOLO Frameworks. Diagnostics.

[10] Fang Y.J., Huang C.W., Karmakar R., Mukundan A., Tsao Y.M., Yang K.Y., Wang H.C. (2024). Assessment of Narrow-Band Imaging Algorithm for Video Capsule Endoscopy Based on Decorrelated Color Space for Esophageal Cancer: Part II, Detection and Classification of Esophageal Cancer. Cancers.

[11] Tang S., Ou J., Liu J., Wu Y.P., Wu C.Q., Chen T.W., Zhang X.M., Li R., Tang M.J., Yang L.Q. (2021). Application of contrast-enhanced CT radiomics in prediction of early recurrence of locally advanced oesophageal squamous cell carcinoma after trimodal therapy. Cancer Imaging.

[12] Xie C., Yang P., Zhang X., Xu L., Wang X., Li X., Zhang L., Xie R., Yang L., Jing Z. (2019). Sub-region based radiomics analysis for survival prediction in oesophageal tumours treated by definitive concurrent chemoradiotherapy. EBioMedicine.

[13] Ococks E., Sharma S., Ng A., Aleshin A., Fitzgerald R.C., Smyth E. (2021). Serial Circulating Tumor DNA Detection Using a Personalized, Tumor-Informed Assay in Esophageal Adenocarcinoma Patients Following Resection. Gastroenterology.

[14] Lambin P., Leijenaar R., Deist T.M., Peerlings J., de Jong E., van Timmeren J., Sanduleanu S., Larue R., Even A., Jochems A. (2017). Radiomics: The bridge between medical imaging and personalized medicine. Nat. Rev. Clin. Oncol..

[15] Liu Z., Wang S., Dong D., Wei J., Fang C., Zhou X., Sun K., Li L., Li B., Wang M. (2019). The Applications of Radiomics in Precision Diagnosis and Treatment of Oncology: Opportunities and Challenges. Theranostics.

[16] Lambin P., Rios-Velazquez E., Leijenaar R., Carvalho S., van Stiphout R.G., Granton P., Zegers C.M., Gillies R., Boellard R., Dekker A. (2012). Radiomics: Extracting more information from medical images using advanced feature analysis. Eur. J. Cancer.

[17] Huang Y., Liu Z., He L., Chen X., Pan D., Ma Z., Liang C., Tian J., Liang C. (2016). Radiomics Signature: A Potential Biomarker for the Prediction of Disease-Free Survival in Early-Stage (I or II) Non-Small Cell Lung Cancer. Radiology.

[18] Hosny A., Parmar C., Coroller T.P., Grossmann P., Zeleznik R., Kumar A., Bussink J., Gillies R.J., Mak R.H., Aerts H. (2018). Deep learning for lung cancer prognostication: A retrospective multi-cohort radiomics study. PLoS Med..

[19] Liu Z., Meng X., Zhang H., Li Z., Liu J., Sun K., Meng Y., Dai W., Xie P., Ding Y. (2020). Predicting distant metastasis and chemotherapy benefit in locally advanced rectal cancer. Nat. Commun..

[20] Tibermacine H., Rouanet P., Sbarra M., Forghani R., Reinhold C., Nougaret S. (2021). Radiomics modelling in rectal cancer to predict disease-free survival: Evaluation of different approaches. Br. J. Surg..

[21] Anconina R., Ortega C., Metser U., Liu Z.A., Elimova E., Allen M., Darling G.E., Wong R., Taylor K., Yeung J. (2022). Combined 18 F-FDG PET/CT Radiomics and Sarcopenia Score in Predicting Relapse-Free Survival and Overall Survival in Patients With Esophagogastric Cancer. Clin. Nucl. Med..

[22] Cui Y., Li Z., Xiang M., Han D., Yin Y., Ma C. (2022). Machine learning models predict overall survival and progression free survival of non-surgical esophageal cancer patients with chemoradiotherapy based on CT image radiomics signatures. Radiat. Oncol..

[23] Yang J., Wu Q., Xu L., Wang Z., Su K., Liu R., Yen E.A., Liu S., Qin J., Rong Y. (2020). Integrating tumor and nodal radiomics to predict lymph node metastasis in gastric cancer. Radiother. Oncol..

[24] Jing Z., Qiang G., Fang H., Zhan-Li L., Hong-An L., Yu S. (2020). A Novel 3D Reconstruction Algorithm of Motion-Blurred CT Image. Comput. Math. Methods Med..

[25] Sun K., Jiao Z., Zhu H., Chai W., Yan X., Fu C., Cheng J.Z., Yan F., Shen D. (2021). Radiomics-based machine learning analysis and characterization of breast lesions with multiparametric diffusion-weighted MR. J. Transl. Med..

[26] Kang J.J., Chen Y., Xu G.D., Bao S.L., Wang J., Ge M., Shen L.H., Jia Z.Z. (2022). Combining quantitative susceptibility mapping to radiomics in diagnosing Parkinson’s disease and assessing cognitive impairment. Eur. Radiol..

[27] Zheng Y., Zhou D., Liu H., Wen M. (2022). CT-based radiomics analysis of different machine learning models for differentiating benign and malignant parotid tumors. Eur. Radiol..

[28] Tan Y., Feng L.J., Huang Y.H., Xue J.W., Long L.L., Feng Z.B. (2024). A comprehensive radiopathological nomogram for the prediction of pathological staging in gastric cancer using CT-derived and WSI-based features. Transl. Oncol..

[29] Qiu Q., Duan J., Deng H., Han Z., Gu J., Yue N.J., Yin Y. (2020). Development and Validation of a Radiomics Nomogram Model for Predicting Postoperative Recurrence in Patients with Esophageal Squamous Cell Cancer Who Achieved pCR After Neoadjuvant Chemoradiotherapy Followed by Surgery. Front. Oncol..

[30] Xie C., Yu X., Tan N., Zhang J., Su W., Ni W., Li C., Zhao Z., Xiang Z., Shao L. (2024). Combined deep learning and radiomics in pretreatment radiation esophagitis prediction for patients with esophageal cancer underwent volumetric modulated arc therapy. Radiother. Oncol..

[31] Gong J., Zhang W., Huang W., Liao Y., Yin Y., Shi M., Qin W., Zhao L. (2022). CT-based radiomics nomogram may predict local recurrence-free survival in esophageal cancer patients receiving definitive chemoradiation or radiotherapy: A multicenter study. Radiother. Oncol..

[32] Cui J., Li L., Liu N., Hou W., Dong Y., Yang F., Zhu S., Li J., Yuan S. (2023). Model integrating CT-based radiomics and genomics for survival prediction in esophageal cancer patients receiving definitive chemoradiotherapy. Biomark. Res..

[33] Taciuc I.A., Dumitru M., Vrinceanu D., Gherghe M., Manole F., Marinescu A., Serboiu C., Neagos A., Costache A. (2024). Applications and challenges of neural networks in otolaryngology (Review). Biomed. Rep..

