# Supplementary material for: Multi-Centered Pre-Treatment CT-Based Radiomics Features to Predict Locoregional Recurrence of Locally Advanced Esophageal Cancer After Definitive Chemoradiotherapy
Source: Cancers (Basel). 2025 Jan 3;17(1):126. doi: 10.3390/cancers17010126 (PMC11720276; doi:10.3390/cancers17010126)
Supplement: Supplementary file 1 [file cancers-17-00126-s001.zip › cancers-3295805-supplementary.pdf]

Supplementary table S1. Distribution of seleted radiomics features

Feature types	Number of selected radiomics features				
	Training set 1	Training set 2	Training set 3	Training set 4	Training set 5
HOG features	16	16	16	13	16
Texture features	1	3	2	2	0
Wavelet features	3	2	3	4	4
Statistics features	10	9	9	11	10
